# Death-associated protein kinase: A molecule with functional antagonistic duality and a potential role in inflammatory bowel disease (Review)

**DOI:** 10.3892/ijo.2015.2998

**Published:** 2015-05-11

**Authors:** SARA STEINMANN, KRISTINA SCHEIBE, KATHARINA ERLENBACH-WUENSCH, CLEMENS NEUFERT, REGINE SCHNEIDER-STOCK

**Affiliations:** 1 Experimental Tumorpathology, FAU Erlangen-Nürnberg, 91054 Erlangen, Germany; 2 Institute of Pathology, FAU Erlangen-Nürnberg, 91054 Erlangen, Germany; 3 Department of Medicine 1, University Hospital Erlangen, FAU Erlangen-Nürnberg, Kussmaul Campus for Medical Research, 91052 Erlangen, Germany

**Keywords:** inflammatory bowel disease, death-associated protein kinase, colorectal carcinogenesis

## Abstract

The cytoskeleton-associated serine/threonine kinase death-associated protein kinase (DAPK) has been described as a cancer gene chameleon with functional antagonistic duality in a cell type and context specific manner. The broad range of interaction partners and substrates link DAPK to inflammatory processes especially in the gut. Herein we summarize our knowledge on the role of DAPK in different cell types that play a role under inflammatory conditions in the gut. Besides some promising experimental data suggesting DAPK as an interesting drug target in inflammatory bowel disease there are many open questions regarding direct evidence for a role of DAPK in intestinal inflammation.

## 1. Introduction

Phosphorylation of proteins by kinases is the most frequent protein modification and plays a key role in multiple signal transduction pathways in normal and cancer cells. In recent years protein kinases have become novel promising candidates for targeted anticancer therapy. To identify and characterize kinases as biomarkers for tumor transformation or progression is a major challenge for clinicians, oncologists, and molecular biologists. The cytoskeleton-associated serine/threonine kinase death-associated protein kinase (DAPK) has been described as a cancer gene chameleon showing functional antagonistic duality in a cell type and context specific manner (1). Cancer genes are classified according to whether they function in a dominant or recessive manner. Dominant cancer genes (oncogenes) are constitutively activated by gain of function mutations and stimulate cell growth and survival. For recessive genes (tumor suppressors) the loss of function leads to the inactivation and loss of cell cycle control and repair capacity. Mutations in the DAPK gene are very rare. There are many other mechanisms such as promoter hypermethylation, autophosphorylation of calmodulin-domain, protein degradation or inhibitory phosphorylations of the DAPK molecule itself that might inactivate DAPK. Noteworthy, DAPK can act not only through its catalytic activity but also triggers multiprotein complexes through its scaffold function (2). The broad regulation levels of this kinase let it be involved in many different cellular functions such as cell death (apoptosis, anoikis, autophagy), repair and mechanosensing (3–5).

Colorectal cancer (CRC) develops in a multistep process and specific molecular hits have been defined that are closely correlated with single morphological alterations along the carcinogenesis process summarized in the Vogelstein model (6). For sporadic cancer two major different pathogenetic pathways exist: the chromosomal instability phenotype (CIN, counts for 85% of tumors) and the microsatellite instability phenotype (MSI, counts for 15% of tumors), both are caused by loss of a general genetic stability. In 2012 a three-group classification system has been reported according to alterations in known signal transduction pathways: i) WNT and TGFβ signaling ii) PIK3CA and RAS signaling, and iii) p53 signaling (7,8). Also epigenetic alterations contribute to altered gene expression in colorectal cancer (9). In this regard a CpG island methylator phenotype has been described (CIMP). Moreover, CIMP is included in different molecular classification systems. Nevertheless its prognostic predictive role is not clarified due to lack of unified test systems. There are also other risk factors for the development of a colorectal carcinoma. Patients with inflammatory bowel disease (IBD) show an increased risk for tumor development. The pathogenesis of IBD-associated carcinogenesis is poorly understood. What we know is that similar to sporadic cancer also IBD-associated cancer is a consequence of a sequence of single molecular alterations (10).

Although it is not surprising that many molecular hits are overlapping in both cancers the major difference is the frequency and the timing of these molecular alterations (10,11). For DAPK in sporadic colorectal cancer there is a loss of protein by promoter hypermethylation already in very small tumors and thus DAPK loss plays a role at very early steps of the tumor formation process (12). Moreover, loss of DAPK in colorectal carcinomas has been associated with higher lymph node metastasis and poor prognosis (13). In contrast, besides an early inactivation by promoter methylation in a subset of tumors, DAPK is remarkably activated in colon cancer in the setting of inflammation (14). So far only one of the two major IBD forms has been studied for DAPK expression: ulcerative colitis (UC) (15). There are no data on the role of DAPK in Crohn’s disease (CD). Recently, it has been shown that DAPK may play a role in UC-associated tumor transformation (16). Pro- as well as anti-inflammatory functions have been suggested for DAPK, dependent on the cell type and stimulus.

As the development of colorectal cancer is a long-term complication of chronic inflammation it would be helpful for patient management to identify molecular biomarkers that predict the risk of tumor development as early as possible. DAPK might be a possible candidate for therapeutic intervention but its gene chameleon nature needs an ultimate understanding of its functions and regulation in different cell types under different inflammatory stimuli.

## 2. DAPK structure and functional domains

**Figure 1:**
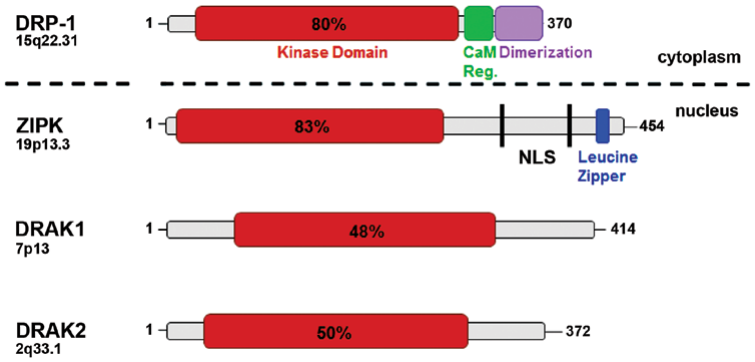
Structure of different isoforms and functional domains of DAPK family members. Percentage gives structural similarity to the DAPK molecule.

DAPK1 (here referred as DAPK) represents one of the five members of the DAPK family (4). These molecules differ in size and subcellular localization (Fig. 1, Tables I–III). DAPK-related protein 1 (DRP-1, DAPK2) and zipper-interacting protein kinase (ZIPK, DAPK3) share the highest homology with approximately 80% at the N-terminus whereas DAPK related apoptosis inducing kinase 1 and 2 (DRAK1, DRAK2) have only 50% homology compared with DAPK (17–20). In contrast to the cytoskeleton-associated family members, ZIPK has also nuclear functions and interacts with transcription factors such as ATF4 and STAT3 (21). DAPK is a multi-domain structure protein that exerts its action through the catalytic activity and phosphorylation of specific substrates with DAPK-containing motifs or as a scaffold protein by stabilizing or triggering multiprotein complexes. One of the most important functional domains is the calmodulin auto-regulatory domain that is localized inside of the catalytic cleft. Calmodulin binding leads to changes in conformation which allow DAPK activation via binding to its substrates (22). Also auto-phosphorylation at residue Ser308 inhibits the DAPK function by reducing its affinity to calmodulin (23).

Besides the prominent function of the catalytic subunit all additional domains such as the ankyrin repeats, the ROC-COR domain in the cytoskeleton-binding region, and the death domain have particular function in the concerted action of this multifunctional protein (Tables I and II). DAPK contains 8 ankyrin repeats that determine primarily the localization of DAPK. Moreover, this region is important for protein-protein interactions. Thereby a negative DAPK regulator, the DAPK-interacting protein (DIP1), is binding at the ankyrin repeat domain. Phosphorylation of Tyr491/Tyr492 by Src tyrosine kinase within the ankyrin repeat domain leads to inactivation of DAPK (24) whereas the interaction with the phosphatase LAR reconstitutes the activity of DAPK (24).

Bialik and Kimchi *et al* (25) have shown that DAPK is a member of the ROCO protein family that is characterized by the tandem appearance of the ROC (Ras-like GTPase) domain, and a characteristic COR (C-terminal of Roc) domain. The ROC domain overlaps with the cytoskeleton binding region of DAPK and mediates the GTP binding at the P-loop motif. This led to the definition of DAPK as a GTP-binding protein with intrinsic GTPase activity whereby GTP binding negatively regulates DAPK’s activity. The serine/threonine protein phosphatase PP2A, the only known phosphatase dephosphorylating the inactive pDAPK^Ser308^, also interacts with the ROC-COR domain of DAPK (26).

The death domain at the DAPK C-terminus mediates DAPK’s function in Fas- and TNF-induced cell death (3). It interacts with microtubule affinity regulating kinases (MAPK1/2) that phosphorylate tau and thereby destabilize microtubules (27). ERK is known to phosphorylate DAPK at Ser735 in the death domain increasing its catalytic activity. The death domain-mediated phosphorylation of the TSC2 protein leads to autophagy induction (28). Also the interaction of the transmembrane receptor UNC5H is mediated through the death domain. UNC5H recruits DAPK and PP2A to the lipid rafts where Ser308 is then dephosphorylated leading finally to an increase in activity. There are death domain-mediated interactions that result in a destabilization of DAPK such as the binding with KLHL20, an adaptor for the Cullin3 ligase, which promotes the proteasomal degradation of DAPK.

## 3. Regulation of DAPK

The cellular level of DAPK can be regulated manifold. On the transcriptional level promoter hypermethylation has been described that strongly correlates with DAPK protein loss (21,29,30). The promoter of DAPK has a high density of CpG islands and motifs for a number of transcription factors are located within these regions such as for NFκB, E2F1 or AP1 (21). For colon tumors, the literature reports a wide range of 5–80% methylation frequency possibly caused by investigating different CpG islands in different studies. So far there is no systematic study comparing the significance of different CpG islands for protein expression. Despite the high frequency of hypermethylated tumors DAPK is not included in the CIMP phenotype gene panel.

DAPK can be transcriptionally inhibited by the pro-inflammatory transcription factors STAT3 and NFκB (16,31,32). In addition, DAPK mRNA expression can be triggered by p53 (33), C/EBP-β (34), HSF1 (35), and SMAD (36). Whereas C/EBP-β binding depends on IFNγ exposure, the binding of SMAD to the corresponding motifs is triggered by TGF-β. In general, DAPK might be upregulated transcriptionally in response to DNA damage (21,37).

Jin and Gallagher (38) identified a second DAPK transcript that is alternatively spliced via intron retention which leads to the inclusion of a new stop codon downstream. Therefore, the alternative transcript is extended by 30 bp. Of note, both transcripts encode proteins with different cellular functions whereby DAPKα (classical DAPK1) is pro-apoptotic and DAPKβ is pro-survival.

Recently, miRNAs (miR-103, miR-107) have been identified to target DAPK 3′UTR. High miR-103 and miR-107 expression was correlated with high level of metastases and poor survival in colorectal cancer patients which is in agreement with DAPK’s role as a metastasis suppressor (13). A data base search *in silico* predicted also some additional miRNAs that might play a role in DAPK regulation (21). However, experimental evidence for these miRNAs is lacking.

The stability of DAPK is regulated post-translationally by two different intracellular proteolysis systems (Table III). One is the ubiquitin proteasome system with HSC70-interacting protein (CHIP) that forms the complex between DAPK and HSP90 (39), DIP1 that interacts with the ankyrin repeat domain of DAPK (40) or the KLHL20 protein that acts as an adaptor for Cullin3-based E3-ligases and interacts with the death domain of DAPK (41). Several reports show that selective mechanisms exist for reducing cellular DAPK levels by directed targeting degradation of active DAPK (39). The other degradation system is the autophagocytic/lysosomal system. Here, the tuberous sclerosis complex (TSC) formed by its two proteins TSC1 (hamartin) and TSC2 (tuberin) inhibits the activation of mammalian target of rapamycin complex 1 (mTOR). Binding of the death domain to TSC2 leads either to phosphorylation of TSC2 by DAPK, a dissociation of the complex and mTOR activation or a reduction in DAPK levels directly by TSC2 via a post-translational mechanism (42). Finally, there is a non-ubiquitin, non-autophagic pathway for DAPK regulation which is dependent on cathepsin B. Cathepsin B binds to C-terminus region between the cytoskeleton-binding domain and the death domain and leads to a decrease in DAPK expression (43).

## 4. DAPK interactome and substrates

In addition to the multitude of DAPK upstream regulators controlling its catalytic activity via phosphorylation events and also its structural stability as mentioned above, further DAPK-binding proteins grouped as the DAPK interactome have been discovered (44) (Tables I and III). Only the minority of these interaction partners has been identified to be DAPK substrates. Thus, it is assumed that the specific binding of these proteins itself to certain consensus DAPK phosphorylation motifs (45) might be sufficient to trigger functional DAPK signaling. Effects of binding of CaM, cathepsin B, DIP1, Hsp90, LAR, Src, TSC2 and UNC5H2 to DAPK were discussed above.

Localized to the actin cytoskeleton, DAPK prominently interacts with cytoskeleton-associated proteins. In 2010, Ivanovska *et al* found first that DAPK has a scaffold function to the LIMK/cofilin complex under TNF treatment which indicates a novel cytoskeleton-associated mechanism of TNF-induced DAPK-dependent actin remodeling and apoptosis in colorectal cancer cells (2). Henshall *et al* demonstrated that the interaction of actin and TNFR-1 with DAPK in rat brain is involved in the recruitment of DAPK to cell death signaling complexes including TNFR-1 and another DAPK interaction partner FADD (46). Actin binding was shown to cause structural rearrangement of microfilaments. Further on, they suggest that 14-3-3 binding modifies DAPK effects in epileptic brain injury. MAP1B was identified as a positive cofactor in DAPK-mediated autophagy including vesicle formation and membrane blebbing. In addition, beclin-1 activation by DAPK and further protein-protein interaction also was found to trigger autophagy (47). ERK enhances death-promoting effects by DAPK Ser735 phosphorylation (48) whereas Ser289 phosphorylation by RSK has a reducing effect on apoptotic activity of DAPK (49). Targeted by PKD, DAPK was found to mediate JNK signaling and caspase-independent cell death upon oxidative stress (50). ZIPK and DAPK were shown to functionally cooperate in causing cell death (51).

A list of DAPK interacting partners and DAPK substrates with consensus DAPK motif (RxxS/T and KR/RxxS/T) is given in Tables I and III. It has to be mentioned that the prediction of DAPK substrates based only on phosphorylation sites is not a sufficient tool since some phosphorylation sites do not represent the specific DAPK motif. Nevertheless, the following proteins have been verified to be DAPK substrates: MLC and tropomyosin-1, MCM3, CaM, P21, P53, S6, Syntaxin-1A (52–58), TAU (MAPT) (27). MLC, ZIPK, Beclin1, HSF1, and tropomyosin-1 link DAPK to cell death associated membrane blebbing, cell motility and stress fiber formation (52,53).

## 5. Immunology and inflammatory responses associated with IBD

Inflammatory bowel diseases (IBD) comprise a heterogeneous set of gastrointestinal (GI) tract disorders which are grouped into two major entities, namely CD and UC (59,60). IBD typically affect children and young adults and the chronically relapsing inflammation of the GI-tract can cause a high individual and socioeconomic disease burden for people suffering from IBD. Despite considerable progress in IBD therapy during the past years, treatment options are still limited and all potent therapeutics bear the risk of relevant side effects, e.g. by suppressing immune effector functions resulting in increased susceptibility to infections.

The precise etiology of IBD has not been clarified yet, but it is well accepted that multiple factors are involved in the pathogenesis. Both CD and UC are characterized by dysregulated immune-responses in genetically predisposed individuals influenced by the microbiome and additional environmental cues (59,60). In addition, defects of the intestinal epithelial barrier might precede and influence the onset of IBD (61).

Recent genetic and immunological investigations have revealed important insights into molecular players involved in the pathogenesis of IBD (62). Genome-wide association studies (GWAS) have linked susceptibility to IBD with single nucleotide polymorphisms (SNP) at more than 150 gene loci which are markedly enriched in genes involved in primary immunodeficiency as well as immune-mediated diseases (63). The contribution of a single risk locus to the individual’s IBD risk appears low, and the majority of these loci is shared by both UC and CD.

Various innate and adaptive immune cells including macrophages, T effector cells, regulatory T cells or innate lymphoid cells have been implicated in intestinal inflammation and IBD pathogenesis (62–64). Moreover, current IBD therapies are primarily directed against imbalanced immune responses in IBD patients with strategies targeting the expansion and/or homing of pro-inflammatory T cell lymphocytes and the T cell-macrophage axis (59,60,65–67). Notably, DAPK is well-known for being involved in modulating pro- and anti-inflammatory immune responses in macrophage and T cell studies suggesting a potential role in IBD (68).

There is also growing evidence that defects of the intestinal epithelial barrier may trigger and influence intestinal inflammation in IBD patients (61). Noteworthy, we were able to demonstrate that DAPK can act as negative regulator of STAT3 in IECs suggesting an important role for DAPK in barrier function and potentially during IBD pathogenesis (16).

Cytokines are central players of the immunological crosstalk between different lamina propria cells and they can also shape barrier function by signaling from immune cell subsets to the intestinal epithelium (69,70). It is well-known that the expression of multiple cytokines is elevated in the intestine during ongoing gut inflammation (59,60,64). In addition, functional studies in experimental models have revealed that cytokines can potently influence the course of intestinal inflammation (59,60,64). Such studies are further supported by genetic evidence from GWAS in IBD patients correlating single nucleotide polymorphisms with DNA loci containing genes associated with cytokine signaling (63).

Tumor necrosis factor alpha (TNF-α) is a pro-inflammatory key molecule promoting the perpetuation of chronic intestinal inflammation in IBD, and anti TNF-α therapies are potent treatment options within current IBD-therapies for a substantial portion of IBD patients (59,60,71). Of note, several studies reported that DAPK is crosslinked with TNF-receptor signaling and NF-κB activation providing further evidence for a potential therapeutic importance of DAPK in IBD. However, it was demonstrated that DAPK can process apparently opposing roles when either inhibiting or promoting inflammation (16,35,68,72–74). Thus, further analyses with careful characterization of cell type specific actions and context-dependent influences are needed before DAPK might be considered a therapeutic target candidate in IBD. Besides TNF-α, several other pro-inflammatory cytokines have been associated with intestinal inflammation (64). Some of them such as IL-1β, IL-6, IL-17 and IL-18 are also known to be modulated by DAPK (16,75–77). In addition, DAPK is required for the formation of the NLRP3 inflammasome which is a key regulator for the expression of pro-inflammatory cytokines including IL-1β and IL-18 by macrophages and has been linked to IBD (75,78,79).

TGF-β receptor signaling is another pathway that seems to play a critical role in IBD. Notably, there is evidence that chronic intestinal inflammation in IBD patients is perpetuated by T effector cells expressing high levels of SMAD7 rendering them less susceptible towards suppression by regulatory T cells and TGF-β signaling (80). Moreover, SMAD7 inhibition by antisense oligonucleotides has evolved as promising therapeutic strategy in patients with CD (81). DAPK is also connected to TGF-β signal transduction via other SMAD-protein family members (63,82,83).

**Figure 2:**
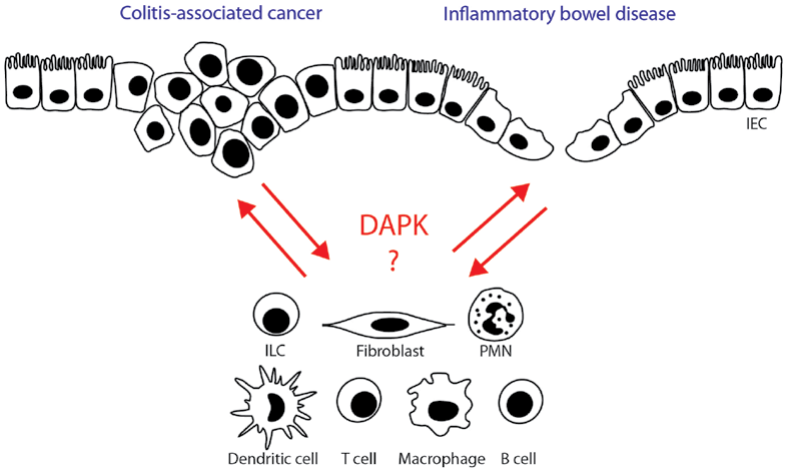
DAPK can be expressed by various cell types including intestinal epithelial cells (IECs) as well as innate and adaptive immune cells typically populating the gut in inflammatory bowel disease (IBD) and colitis-associated cancer (CAC). Further studies are needed to reveal context dependent contributions of DAPK in intestinal cell types potentially modulating the course of intestinal inflammation and/or CAC. Innate lymphoid cell (ILC), polymorphonuclear cells (PMN).

Thus, several lines of evidence suggest that targeting DAPK might influence the course of intestinal inflammation via modulation of immune cell activity and intestinal epithelial barrier function (Fig. 2). However, direct evidence for a critical role of DAPK is limited so far indicating the need for further studies investigating the cell type specific function of DAPK during intestinal inflammation.

## 6. Link of IBD and cancer

DAPK is involved in several forms of cell death including apoptosis, autophagy and anoikis suggesting a potential role in colitis-associated cancer (CAC) (44,84). Although DAPK is often considered a tumor suppressor, pro-survival roles have also been reported (44,84). In particular, there is evidence that DAPK might exert divergent functions under inflammatory conditions (15,16).

IBD patients with longstanding inflammation of the colon are at increased risk for CRC (85). This risk is associated with the duration and anatomic extent of colitis and presence of other inflammatory diseases such primary sclerosing cholangitis (86–88). In fact, recommendations for colon cancer screening (time of initial screening colonoscopy and surveillance intervals) in IBD patients are stricter than for the general population (89).

CRC can be grouped into different entities with sporadic CRC being the most frequent subtype. IBD patients including UC patients as well as CD patients with colonic involvement are particular prone to CAC which can differ from classical sporadic CRC in various features. Sporadic CRC classically develops from normal mucosa via adenomatous polyps to CRC spanning over many years undergoing the adenoma - carcinoma sequence by accumulating sequential gene alterations including APC, KRAS and p53 (90–92). In CAC, similar genetic alterations are found, but a different order of hits including early p53 mutations could pave the way for direct progression to CAC skipping the stage of adenomatous precursor lesions (93,94). CAC can show typical morphological features including flat tumor growth from multiple foci (90). Previous work reported positive feedback mechanisms between DAPK and p53 indicating potential functional relevance of DAPK for CAC growth control (33,95). In addition, our studies have provided direct evidence for the interaction of DAPK with p38 MAPK and STAT3 signaling in inflammation-associated colorectal cancer cells (14,16).

The composition of the local microenvironment can further influence the tumor development. Of note, elevated levels of inflammatory cytokines and growth factors are typically found in CAC allowing for tumor growth promotion in experimental CAC models (96–99). As DAPK modulates the expression and/or signaling of some of these molecules such as IL-1β and IL-18, it is tempting to speculate that DAPK might also influence CAC development by interfering with the proinflammatory cytokine signaling. Moreover, elevated levels of reactive oxygen and nitrogen species typically found with chronic inflammation could further promote DNA damage and DAPK expression during CAC development (21,100).

As in sporadic CRC, epigenetic alterations are often present in CAC and may influence tumor development. DAPK belongs to the genes that are frequent targets of hypermethylation in CRC, and aberrant methylation of DAPK in long-standing UC and CAC was demonstrated (15,101).

In summary, several findings suggest that DAPK could modify molecular mechanisms of colitis-associated tumorigenesis. To date however, it has not been clarified which particular context is critical for rendering DAPK1 either a tumor suppressor or oncogenic molecule in tumor epithelial and/or tumor stromal cells. Upcoming analyses could provide important functional insights and might put DAPK1 on stage as a target for CAC therapy.

## 7. DAPK regulation and function in different cell types of the intestine

**Figure 3:**
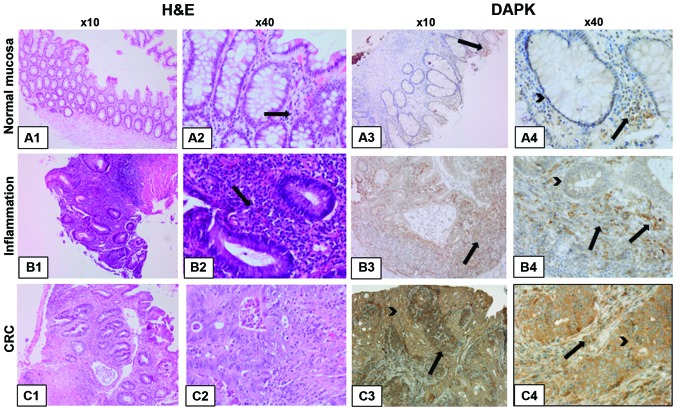
DAPK expression in inflammation (x10 and ×40 fold magnification) (A1 and A2) Example of regular mucosa of the colon with mild inflammation (H&E, →). (A3 and A4) Diffuse expression of DAPK in the cytoplasm of lymphocytes, plasma cells and macrophages in the interstitium. (→) and loss of expression in epithelial cells of colon mucosa (➤). (B1 and B2) Example of severe colitis with crypt architectural distorsion and chronic as well as acute inflammatory cells (H&E, →). (B3 and B4) Strong expression of DAPK in the cytoplasm of inflammatory cells (→). Mild expression in epithelial cells of colon mucosa (➤). (C1 and C2) Example of colorectal carcinoma with intratumoral inflammatory reaction. (C3 and C4) Strong expression of DAPK in the cytoplasm of both, inflammatory cells (→) and tumor cells (➤).

The role of DAPK in the intestine has not been sufficiently elucidated so far. DAPK is expressed and can be activated by numerous cell types including intestinal epithelial cells (IECs) as well as innate and adaptive immune cells typically populating the gut such as macrophages and T cells, respectively. The immunohistochemical DAPK expression in single stages of UC-associated carcinogenesis in regard to different cell types is demonstrated exemplarily in Fig. 3.

Several studies point to a complicated regulatory role of DAPK in IECs (15,16). Noteworthy, DAPK expression in IECs is increased in long-standing UC and correlates with the activity of UC-associated inflammation suggesting a protective role of DAPK during the chronic inflammatory process of UC (15). In addition, DAPK protein expression is elevated in CAC, which may potentially link DAPK to the initiation of the neoplastic process in CAC (15). Remarkably, recent work provided further evidence for a substantial role of DAPK in modulating epithelial cell function (16). In fact, it was demonstrated that STAT3 and DAPK are upregulated in UC but only STAT3 is downregulated in CAC. In addition, DAPK was identified to suppress TNF-induced STAT3 activation and a direct physical interaction of DAPK with STAT3 inducing conformational changes in the STAT3 dimer was proposed as molecular cause (16). Thus, DAPK can act as negative regulator of STAT3 in IECs suggesting an important role for barrier function and regulation of the intestinal homeostasis upon inflammatory stimuli and cancer (16).

DAPK-positive tumor-associated macrophages have been localized in close proximity with apoptotic colorectal cancer cells suggesting direct crosstalk between macrophages and tumor epithelial cells in the intestine (102). Based on studies with purified primary leukocytes and immune cell lines, DAPK might also be involved in the functional regulation of immune cell populations during chronic intestinal inflammation (68). For macrophages, inhibition of inflammation was shown via IFN-γ activated inhibitor of translation (GAIT) complex (103). In addition, macrophages can produce a variety of pro-inflammatory cytokines that are partly controlled by DAPK, e.g. via functional assembly of the NLRP3 inflammasome and activation of caspase-1 (75).

In the adaptive arm of the immune system, DAPK was shown to block the nuclear translocation of ERK1/2 in T lymphocytes (104). Further work demonstrated decreased T cell proliferation and IL-2 production upon stimulation by the T cell receptor (74) indicating that DAPK can interfere with T cell activation which might have important implications for chronic inflammatory diseases such as IBD.

Thus, several pieces of evidence suggest potential contributions of DAPK in the regulation of gut inflammation and intestinal homeostasis. However, further studies are needed to clarify the dominant effects of DAPK in different innate and adaptive immune cell subsets as well as non-immune cells populating the bowel wall during chronic gut inflammation.

## 8. Open questions and future challenges

Regarding direct evidence for a role of DAPK in intestinal inflammation there are many open questions: Which immune cells in the intestinal lamina propria are controlled by DAPK? Are there differential effects on subsets of T helper cell populations including Th1, Th17, and regulatory T cells? What is the role of DAPK in B cells? Are there different effects on macrophage subsets including M1 and M2 macrophages? Which role does DAPK play in non-immune stromal cells such as fibroblasts? How does DAPK interact with signals from the microbiome?

Providing answers to the above questions may help in better understanding of how DAPK controls the function of gut cell populations associated with the pathogenesis of IBD and CRC (Fig. 2). Perspectively, novel insights into molecular disease mechanisms and potential key checkpoints might facilitate the design of new therapeutic approaches for IBD and CRC. Therefore, further studies are awaited that directly reveal the role of DAPK in intestinal homeostasis, intestinal inflammation and CRC.

## Figures and Tables

**Table I:** Interacting partners of DAPK.

Protein	OMIM ID	Binding domain on DAPK
14-3-3	609009	Not defined
Actin	102560	Cytoskeletal-binding region
Beclin-1	604378	Not defined
Calmodulin (CaM)	114180	Calmodulin regulatory domain
Cathepsin B	116810	C-terminal region
DIP1 (MIB1)	608677	Ankyrin repeats
**ERK1/2**	601795/176948	Death domain
Hsp90	140571	Kinase domain
KLHL20	Q9Y2M5 (UniProt)	Death domain
LAR (PTPRF)	179590	Ankyrin repeats
**LIMK/cofilin**	601329/601442	Not defined
MAP1B	157129	Kinase domain
**p38 MAPK**	600289	Not defined
**p53**	191170	Not defined
PP2A	176915	ROC-COR domain
PKD	173900	Not defined
RSK	601684	Not defined
Scr	190090	Ankyrin repeats
**STAT3**	102582	Not defined
**TNFR-1**	191190	Not defined
TSC2	191092	Death domain
UNC5H2	607870	Death domain
ZIPK (DAPK3)	603289	Kinase domain

Bold, inflammation-associated interaction partners of DAPK.

**Table II:** DAPK family - interaction partners of DRP-1, ZIPK, DRAK1 and DRAK2.

Protein	Description	Interacting kinase
RAD1	Cell cycle checkpoint protein RAD1	DRP-1, ZIPK, DRAK1, DRAK2
HORMAD1	HORMA domain containing protein 1	
HORMAD2	HORMA domain containing protein 1	
MAPK1	Mitogen-activated protein kinase 1	DRP-1, ZIPK
MAPK3	Mitogen-activated protein kinase 3	
RAB3IP	RAB3A interacting protein	
MAP2K1	Mitogen-activated protein kinase kinase 1	
UBC	ubiquitin C	ZIPK, DRAK1
MAP2K2	Mitogen-activated protein kinase kinase 2	
RHOV	Ras homolog family member V	DRAK1, DRAK2
MLC1	Megalencephalic leukoencephalopathy with subcortical cysts 1	DRP-1
TGFBR1	Transforming growth factor, β receptor 1	
CSNK1A1	Casein kinase 1, α1	
CSNK1E	Casein kinase 1, ɛ	
NKD1	Naked cuticle homolog 1 *(Drosophila)*	
DAPK2	Death-associated protein kinase 2, DRP-1	
CAMK2A	Calcium/calmodulin-dependent protein kinase II α	
MAP2K5	Mitogen-activated protein kinase kinase 5	
DAXX	Death-domain associated protein	ZIPK
ATF4	Activating transcription factor 4	
AATF	Apoptosis antagonizing transcription factor	
PRKCZ	Protein kinase C, zeta	
UBE2D3	Ubiquitin-conjugating enzyme E2D 3	
MET	Hepatocyte growth factor receptor, proto-oncogene C-Met	
PAWR	PRKC apoptosis WT1 regulator protein	
CDKN1A	Cyclin-dependent kinase inhibitor 1A, p21	
GRB14	Growth factor receptor-bound protein 14	
TPM1	Tropomyosin 1 (α)	
UBE2D1	Ubiquitin-conjugating enzyme E2D 1	
GRB2	Growth factor receptor-bound protein 2	
STAT3	Signal transducer and activator of transcription 3	
UBE2D2	Ubiquitin-conjugating enzyme E2D 2	
DAPK3	Death-associated protein kinase 3, ZIPK	
IRF2BPL	Interferon regulatory factor 2 binding protein-like	
TPM4	Tropomyosin 4	
UNC5B	Unc-5 homolog B *(C. Elegans)*	
GRIN1	Glutamate receptor, Ionotropic, N-methyl D-aspartate 1	
UBE2D4	Ubiquitin-conjugating enzyme E2D 4	
AK3	Adenylate kinase 3	
RPL34	Ribosomal protein L34	
TCP10L	T-complex 10-Like	
RAD21	RAD21 homolog (S. Pombe)	
PSMC3IP	PSMC3 interacting protein	
ATM	Ataxia telangiectasia mutated	
ULK3	Unc-51 like kinase 3	
HDAC3	Histone deacetylase 3	DRAK1
CHP1	Calcineurin-like EF-hand protein 1	DRAK2
RPS24	Ribosomal protein S24	
CHEK2	Checkpoint kinase 2	

**Table III:** DAPK substrates.

Protein	OMIM ID	Phosphorylation consensus site (Consensus: KRxxxxxKRRxxS/T)
Beclin-1	604378	RLKVT^119^GDL
CaM	114180	GSRREERSLS^115^APG
MCM3	602693	TKKTIERRYS^160^DLTTL
MLC	609211	TTKKRPQRATS^19^NVF
p21	116899	RKRRQT^145^SMTDFYHSK
p53	191170	PPLSQET^18^FS^20^DLWKLL
S6	180460	QIAKRRRLS^235^SLRAS
Syntaxin-1A	186590	IIMDSSIS^188^KQALSEIE
Tau (MAPT)	157140	(1)
Tropomyosin-1	191010	HALNDMTS^283^I
ZIPK (DAPK3)	603289	KT^299^TRLKEYTIKS^309^HS^311^S^312^LPPNNS^318^YADFERFS^326^
**HSF-1**	140580	YSRQFS^230^LE…DERPLS^290^SS…PGRPSS^320^VD…RGHT^355^DTEGRPPS^363^PP

Mentioned indications are summarized from Stevens *et al* (28), Bialik and Kimchi (4), Benderska and Schneider-Stock (1), and Ivanovska *et al* (2). Bold, Inflammation-associated interaction partners of DAPK.
